# A Systematic Review of International Evidence to Inform Occupational Therapy Practice Guidelines for Total Knee Replacement in the Korean Health System

**DOI:** 10.1155/oti/5583913

**Published:** 2026-04-02

**Authors:** Junghun Aj Kim, Seong-Kyu Ha, Min-Ye Jung

**Affiliations:** ^1^ Department of Occupational Therapy, College of Health Science, Kangwon National University, Chuncheon-si, Republic of Korea, kangwon.ac.kr; ^2^ Department of Occupational Therapy, College of Health and Biology, Semyung University, Jecheon-si, Republic of Korea, semyung.ac.kr; ^3^ Department of Occupational Therapy, College of Software and Digital Healthcare Convergence, Yonsei University, Wonju-si, Republic of Korea, yonsei.ac.kr

## Abstract

**Introduction:**

This review synthesizes international evidence on occupational therapy (OT) interventions for total knee replacement (TKR) as part of the project “Developing Occupational Therapy Practice Guidelines for Total Knee Replacement,” with the goal of informing practice within the Korean healthcare system. It evaluates OT interventions’ effectiveness in enhancing recovery pathways amid an aging population, increasing healthcare costs, and the need for patient satisfaction and recovery.

**Method:**

Centering on OT interventions in Korea for adults with post‐TKR, this review adhered to the Royal College of Occupational Therapists′ guidelines and PRISMA standards and searched nine electronic databases for literature published between 2000 and November 2021. A broad stakeholder group, including medical professionals and social workers, contributed to refining the research scope of outcomes.

**Results:**

From an initial pool of 10,749 articles, 51 studies were selected, predominantly cohort studies. Analysis identified key‐OT interventions improving activities of daily living, mental health, community reintegration, patient satisfaction, and notably reducing hospital stay lengths.

**Conclusion:**

This review highlights the multifaceted contributions of OT in TKR rehabilitation, with benefits observed across physical, mental, and cognitive recovery. It supports the need for policy changes to broaden the role of OT. Future research, particularly high‐quality and longitudinal studies, is warranted to further strengthen the evidence base and inform the ongoing development of OT practice across diverse healthcare systems.

## 1. Introduction

Arthritis of the knee is a growing global health challenge that significantly reduces the quality of life for millions worldwide [1]. From 2005 to 2015, the incidence of knee arthritis escalated by 32.9%, highlighting an alarming trend [2]. As of 2020, approximately 654 million individuals globally are affected, with 8.67 million new cases each year [3].

In response to this burden, particularly among older adults, total knee replacement (TKR) has become a key intervention for reducing pain and restoring function when less invasive treatments are insufficient [4]. Demand for TKR has risen markedly, with over 70,000 procedures performed annually in the United Kingdom and approximately 1.37 million in the United States, alongside similar trends in other countries [4–6].

As the volume of TKR procedures has increased, the associated national healthcare cost burden has grown, leading to the adoption of evidence‐based care pathways aimed at improving postoperative function and reducing hospital stay. In many Western healthcare systems, these approaches have reduced postoperative hospitalization to approximately 1–2 days in the United States [5]. However, unlike many Western systems, South Korea faces a distinct challenge. Despite performing over 70,000 TKR procedures annually, largely due to population aging, the average postsurgical hospital stay remains substantially longer at 19.4 days [7]. This prolonged hospitalization not only affects patient recovery but also contributes to higher medical costs, placing TKR‐related care among the major healthcare expenditures in South Korea [7, 8].

Reducing hospitalization time and enhancing recovery outcomes have become key priorities, and occupational therapy (OT) plays a critical role in achieving these goals. OT interventions for joint replacement patients have been shown to shorten hospital stays, lower costs, reduce readmissions, improve postdischarge function, expedite recovery, enhance patient satisfaction, and support mental health [9–14]. For this reason, OT services for TKR patients are implemented in many countries.

In South Korea, however, despite 19,136 registered occupational therapists (38.8 per 100,000 population), orthopedic OT capacity remains structurally limited. Only 18 of 171 respondents (10.5%) reported experience working in orthopedic departments, OT for orthopedic surgery patients is primarily prescribed by rehabilitation medicine rather than orthopedic surgery (74.7% vs. 4.8% by orthopedic surgery), and insurance reimbursement outside rehabilitation medicine is restricted to OT categories with relatively low fees, constraining service intensity and OT employment in orthopedic settings [15].

For these reasons, many patients in Korea rarely receive OT services after TKR despite growing evidence, reflecting persistent systemic barriers [15, 16]. To address this gap, previous research has emphasized the need for evidence‐based OT practice guidelines tailored to the Korean healthcare system [15, 17]. Although international reviews have examined OT or multidisciplinary rehabilitation for TKR, this study is the first to synthesize global evidence to inform OT practice guidelines in Korea.

Therefore, the aim of this study is to systematically review and evaluate international evidence on OT interventions for adults after TKR, with the purpose of informing OT practice guidelines in Korea.

## 2. Method

As this study involved gathering and incorporating opinions from diverse stakeholders to inform the development of practice guidelines, it was approved by the Yonsei University Mirae Campus Institutional Review Board (1041849‐202006‐BM‐070‐01) prior to commencement.

Our study sought to answer the question: “What evidence exists for OT clinical interventions that can be implemented in Korea for adults who have undergone total knee replacement surgery?” To address this question, we first defined the scope of the guideline, including the inclusions, exclusions, and anticipated outcomes. This process was guided by the *Practice Guideline Development Manual* (4th Edition) of the Royal College of Occupational Therapists [18] to ensure a comprehensive and stakeholder‐informed approach.

Stakeholder input was used to refine the scope of the review, particularly in defining outcome domains and feasibility criteria relevant to the Korean healthcare system, which in turn informed the inclusion criteria and study selection process. Stakeholders included seven orthopedic doctors, three nurses, six physiotherapists, and five social workers, each contributing valuable perspectives to the expected outcomes. Draft expected outcomes, informed by previous systematic reviews on OT for TKR patients, were shared with stakeholders for feedback. The agreed‐upon outcomes were: improvement in activities of daily living (ADL) function, mental health enhancement (particularly reduction of anxiety and depressive symptoms), patient satisfaction, community reintegration, and reduction in hospital length of stay. These outcomes were used to guide the review process.

### 2.1. Study Design

The methodology followed the RCOT guideline development manual and applied the PICO framework to define the research question and inclusion/exclusion criteria (Table 1). The comparison component was not applicable, as this review is aimed at identifying and mapping OT interventions relevant to guideline development rather than to conduct comparative effectiveness analyses. The review employed a predefined search strategy, clearly stated inclusion and exclusion criteria, and independent screening by two reviewers to ensure methodological rigor and transparency.

**Table 1:** Inclusion criteria.

PICO	Inclusion criteria
Population	Adults aged 18 years and over who have undergone total knee replacement. (This demographic was chosen due to the higher prevalence of knee replacement surgeries in this age group and the unique challenges they face in postoperative recovery.)
Intervention	OT interventions (Aimed to explore various OT approaches and their effectiveness in aiding recovery post‐knee replacement.)
Comparison	“Comparison” component was not applicable in this context.

Outcomes	Activities of daily living function
	Mental health (specifically, anxiety, and depressive symptoms)
	Patient satisfaction
	Reintegration into the community
	Length of hospital stay

### 2.2. Electronic Search

The literature search for this systematic review was conducted over an 18‐month period (May 2020 to November 2021) using a structured strategy developed with a university librarian. The extended search period reflected iterative refinement of search terms and database selection, incorporation of stakeholder input, and delays caused by COVID‐19. Although initiated by the first author, the search strategy was reviewed and refined in close collaboration with the coauthors and the librarian throughout key stages such as search term development and database selection. Screening and data extraction were conducted independently by two reviewers to minimize bias.

We targeted literature published from 2000 to November 2021 to capture two decades of development in OT interventions for knee replacement. Major databases included PubMed, Medline, CINAHL, EMBASE, and Web of Science. Specialist resources were also searched, such as OTDBASE, OT SEARCH, OTseeker, and the Cochrane Library of Systematic Reviews. In addition, we reviewed guidelines and publications from authoritative bodies including NICE, SIGN, AHRQ, and GIN.

Keywords aligned with stakeholder‐identified outcomes included:

• “knee replacement”
• “rehabilitation”
• “occupational therapy”
• outcome terms such as “ADL”, “mental health”, “patient satisfaction”, “reintegration into the community”, and “length of hospital stay”

Search techniques included the use of MeSH terms, Boolean operators, proximity operators, truncation, and wildcards to maximize retrieval.

### 2.3. Criteria for Considering Studies for This Review

The inclusion criteria were designed to identify studies involving adults (aged 18 and above) who underwent TKR and received OT interventions targeting the expected outcomes of this guideline. Studies were eligible if OT was provided either as a standalone intervention or as part of a multidisciplinary approach (e.g., combined with physiotherapy, nursing, or patient education). Studies were excluded if they focused solely on other disciplines such as physiotherapy or nursing without any involvement of OT, involved populations under 18, addressed unrelated medical conditions, were not in English, or concentrated only on surgical techniques.

In addition to primary studies, systematic reviews were also included to support the development of practice guidelines grounded in the highest level of available evidence. To avoid the risk of duplicate inference, particularly when individual studies appeared both independently and within systematic reviews, we used the reviews primarily to identify overarching intervention concepts and broader patterns rather than relying on their pooled results.

Studies were excluded if they met any of the following conditions:

• Grey literature (e.g., dissertations, reports, and conference abstracts) or nonpeer‐reviewed publications (e.g., expert opinions)
• Published in languages other than English
• Abstract‐only publications without full‐text availability
• Studies not involving adult TKR patients
• Studies without OT‐related intervention or without sufficient intervention description
• No outcome data or outcomes irrelevant to the review objectives
• Inadequate study designs, such as purely descriptive reports or simple reviews

### 2.4. Data Extraction and Management

Bibliographic data were managed using EndNote, with duplicates removed through automatic detection and manual verification.

After removal of duplicate records, 4203 studies were screened at the title and abstract level by two reviewers (Author 1 and Author 2) using criteria based on the PICO framework. Studies unrelated to TKR, focused on other diseases or disabilities, or not involving intervention‐based research were excluded through discussion. When necessary, a third reviewer was consulted. This process resulted in the selection of 112 studies for full‐text review. Screening decisions were guided by the aim of informing OT practice guideline development.

Two reviewers then independently extracted data from the 112 full‐text articles using a standardized form covering study design, participant characteristics, OT interventions, outcome measures, and key findings. Disagreements were resolved by consensus or, when needed, by consultation with a third reviewer. The study selection process is summarized in the PRISMA flow diagram.

As this review formed part of a guideline development process, quantitative synthesis was not pursued. Instead, the evidence was synthesized narratively to support the identification and mapping of relevant OT interventions.

### 2.5. Risk of Bias Assessment

To assess the methodological quality of the included studies, we used the Critical Appraisal Skills Programme checklists (CASP), which evaluate aspects such as data collection and analysis, potential confounders and biases, transparency, and the accuracy of results. CASP offers different tools for various study designs, enabling us to comprehensively evaluate randomized controlled trials, systematic reviews, case‐control studies, cohort studies, and qualitative research [19].

Following quality appraisal, the evidence level of each paper was categorized according to the GRADE system [18, 19] into four levels: A (very high), B (high), C (low), and D (very low). The initial level could be adjusted upward or downward based on the CASP quality analysis, such as when serious limitations or biases were identified or, conversely, when particularly strong methodological features were present [18]. For example, a retrospective case‐control study using national data or data from large regional hospitals could be upgraded from Grade C to B.

## 3. Results

**Figure 1:**
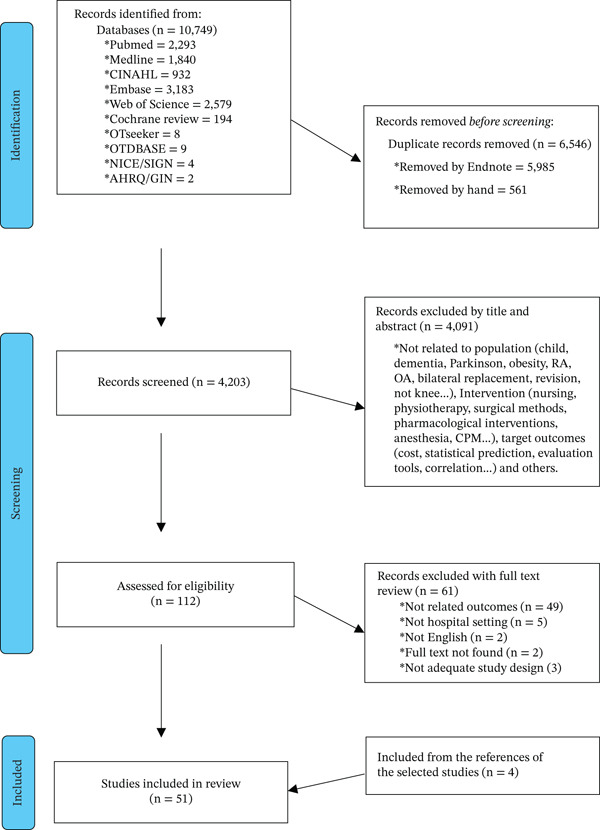
PRISMA diagram studies selection flow.

A total of 10,749 records were identified through database searching. After removal of duplicates and screening of titles and abstracts, 112 articles were assessed for full‐text eligibility. Following full‐text review, 51 studies were included in the final synthesis. The study selection process is presented in Figure 1.

In terms of research design, cohort studies were the most prevalent (*n* = 13), followed by systematic reviews (*n* = 12), clinical trials (*n* = 9), case‐control studies (*n* = 7), case studies (*n* = 4), qualitative studies (*n* = 2), guidelines (*n* = 1), and other study types (*n* = 3). With regard to evidence levels, six studies were classified as Level A, nine as Level B, 31 as Level C, and five as Level D.

Based on the content of the 51 articles, we identified meaningful OT interventions aligned with the stakeholder‐defined expected outcomes. These interventions, along with the corresponding outcome categories and levels of evidence, are summarized in Table 2.

**Table 2:** Summary of individual studies included.

No.	Source	Design/participants	Intervention	Results	CASP	Quality
1	Aasvang, 2015 [20] Denmark	Narrative review	Fast‐track hip and knee arthroplasty	Fast‐track surgical programs reduce cognitive dysfunction by up to 8% compared with 20%–40% in the other settings and shorten hospital stays by 1–3 days compared with nonfast‐track methods	N/A	Grade D Very low
2	Alviar, 2011 [21]	Systematic review	Outcome measurement for hip and knee arthroplasty	A total of 351 (88%) out of 399 items of eight patient‐reported instruments used in THR and TKR were linked to the ICF. Emphasize using the standard outcome measurement.	10/10	Grade B—moderate
3	Amini 2018 [22] United States	Case study TKR	Education, ADL retraining, adaptive equipment provision	Through activity, engagement in occupations, ADL training, education, and adaptive equipment the participant improved his independence and safety with functional mobility and transfers, bathing, toileting, and dressing.	N/A	Grade D—very low
4	Arshad 2014 [23]United Kingdom	Cohort study *N* = 192 THR or TKR	Preoperative education, multimodal analgesia, early mobilization	Enhanced recovery program (OT participated) reduced length of stay (1/2 day shorter) and increased the rate of straight home (96% compared wihth 65%, *p* < 0.01)	8/12	Grade C—low
5	Auyong 2015 [24] United States	Cohort study (retrospective) TKR = 252	Updated enhanced recovery after orthopedic surgery (ERAS) (ERAS) pathway	ERAS (OT participated) reduced length of stay from 76.6 h (3.2 days) to 56.1 h (2.3 days) (*p* < 0.001) (Control: April 2012 to November 2012; intervention: early to July 2013)	10/12	Grade C—low
6	Aydin 2015 [25]	Systematic review THR or TKR	Preoperative education	Preoperative patient education significantly reduces preoperative anxiety.	9/10	Grade B—moderate
7	Baker 2012[26]	Systematic review TKR	Preoperative rehabilitation	Preoperative rehabilitation for patients undergoing TKR is beneficial in reducing the length of hospital stay.	9/10	Grade B—moderate
8	[27] Netherlands	Clinical trial (before and after trial) THR = 98, TKR = 62 (JRP = 78, control = 82)	Total hip or knee replacement joint recovery program (JRP)	JRP reduced in hospital length of stay (reduction of 6.9 day (JRP 4.87days and usual care 11.75days, *p* < 0.001) and improved in functional level.	9/11	Grade C—low
9	Caracciolo and Giaquinto [28] Italy	Cohort study (prospective) TKR = 36 or THR = 36	Self‐perceived distress and self‐perceived functional recovery after postoperative rehabilitation	The comparison between depressed versus nondepressed patients on WOMAC gain score showed significant differences for TKA patients (WOMAC‐pain: *U* = 2.117, *p* < 0.05; WOMAC‐function: *U* = 2.683, *p* < 0.01), thus indicating a better outcome without depression	10/12	Grade C—low
10	Cook 2008 [29] United States	Case study TKR = 74	Joint replacement program (JRP)	The comprehensive JRP (OT participated) for TKA was associated with satisfactory clinical outcomes, short lengths of stay, a high percentage of patients discharged home with outpatient PT, and minimal complications.	N/A	Grade D—very low
11	Coudeyre 2007 [9]	Systematic review	Rehabilitation preoperatively for THR or TKR	Preoperative rehabilitation has benefit in terms of length of the stay and destination at discharge	9/10	Grade B—moderate
12	[30] United States	Randomized controlled trial *N* = 133 (THR or TKR)	Prearthroplasty rehabilitation versus usual care	Prehabilitation group showed lower presurgery anxiety (state anxiety inventory; *R* = 40.0 vs. UC = 45.7) and had a significantly shorter average hospital stay (*R* = 6.55 days vs. UC = 10.5 days, *p* = 0.032)	9/11	Grade B—moderate
13	de Groot et al. (2008)[31] Netherlands	Cohort study	Total hip or knee replacement	A total of 0.7% improvement in actual physical activity (*p* = 0.03) and self‐reported activity increased by 86%—discrepancy between self‐report and measured activity. In contrast to the large effect on pain and stiffness, patients′ capacity, and their self‐reported physical functioning, the improvement in actual physical activity of our patients was less than expected 6 months after surgery.	11/12	Grade C—low
14	Dorsey and Bradshaw, 2017 [10]	Systematic review	Occupational therapy interventions	Multidisciplinary rehabilitation (education, ADL training, joint protection strategies, adaptive equipment training, functional mobility training) is effective for LE joint replacement.	10/10	Grade A—high
15	Dwyer et al. [32] United Kingdom	Case‐control study TKR = 112 (ER = 57, control = 55)	Enhanced recovery (ER) program	Enhanced recovery program (OT participated) are significantly reduced the length of hospital stay (6 vs. 7.8 days, *p* = 0.0003)	10/11	Grade C low
16	Featherall et al. [33] United States	Case‐control study TKR TKR(Care pathway group(2015)=2,321, historical group(2013)=2,328)	Evidence based TKR care pathway	Care pathway reduced length of stay from 3.39 (1.28) days to 2.60 (1.31) days (26.966% reduction, *p* < 0.001) and increased in discharges to home (odds ratio [OR] 3.838) (control group [2013]; intervention group [2015])	10/11	Grade C—low
17	Feng et al. [34]	Review	Multidisciplinary approach for TKR	Multidisciplinary (OT participated) approach reduces LOS and enhances satisfaction of care.	N/A	Grade C—low
18	Gillen et al. [35] United States	Case study TKR = 72 or THR = 35	Occupational therapy community reintegration program	Participants reported significant increases in measures of satisfaction of performance and confidence related to performance and community skills (car, obstacle, appointment, shopping, safety) demonstrated significantly high scores(pre–post, *p* < 0.0001)	N/A	Grade D—low
19	Gleicher et al. (2021) [36] Canada	Clinical trial (before‐and‐after design) TKR = 615 (Intervention = 232, control = 383)	Enhanced recovery after surgery (ERAS) bundle (OT participated)	ERAS (OT participated) decreased LOS from 2.82 to 2.13 days, rehab needs by 10% and 30‐day emergency department visits following discharge by 5%. (Control: January 1 to December 2017; intervention: January 1 2018, to August 31, 2019)	9/11	Grade C—low
20	Grotle et al. [37] Norway	Case study (longitudinal observational study) *N* = 173 (THR = 126, TKR = 46)	Team rehabilitation care (OT participated)	The patients had large improvements in the outcome measures and reported high satisfaction with the care and its organization. (This study describes current practice of team rehabilitation care in terms of structure, process, and outcome for patients and emphasizes OT involvement)	N/A	Grade D—low
21	Harding et al. (2014)[38] Australia	Cohort study (measured from preoperatively to 6 months post operatively) THR = 19, TKR = 25	Physical activity measured via an accelerometer (activity monitor) which was to be worn for a minimum of 10 h a day (during waking hours) over 7 consecutive days	Significant improvements in self‐reported pain during activity (*p* < 0.001), physical function (*p* < 0.001), quality of life (*p* < 0.001) and in physical activity measured by the UCLA activity score (*p* < 0.001).	N/A	Grade C—low
22	Herbold et al. (2011)[39] United States	Case‐control study *N* = 204 (IRF = 102, SNF = 102) (hip fx = 12*%*, THR = 35*%*, TKR = 52*%*)	Intervention=Inpatient rehabilitation facility (IRF) Control=Skilled nursing facility (SNF)	IRF group showed less length of stay (10.74 vs. 25.47), were more likely to ambulate independently (87.5% vs. 74.0%; P.019), managed stairs independently (68.4% vs. 34.7%; P.001), required less home care (33.7% vs. 76.4%; *p* 0.001), and were less likely to use a walker at discharge (41.7% vs. 67.7%; *p* 0.001).	10/11	Grade C—low
23	Huang et al. [40] Taiwan	Randomized controlled study TKR = 243 (Intervention = 126, control = 117)	A total of 40‐min preoperative home rehabilitation education program 4 weeks prior to TKR	LOS was 7 ± 2 (range from 5 to10) days for the study group and 8 ± 1 (range from 5 to 12) days for the control group (CI: −0.795 to‐0.044; *p* = 0.027).	10/11	Grade B—moderate
24	Husted et al. (2008)[41] Denmark	Cohort study (prospective) *N* = 712 THR = 370, TKR = 342	Identify patient characteristics associated with LOS and patient satisfaction after THR or TKR at specialized fast‐track joint replacement unit.	Women had almost 40% greater probability of staying more than 3 days than men and after implementation of the fast‐track procedures, mean LOS was reduced from 8 to 3.8 days	9/12	Grade C—low
25	Isaac et al. [42] United Kingdom	Controlled trial TKR = 130 (Intervention = 50, control = 80)	Accelerated postoperative rehabilitation protocol	Length of stay reduced to 3.6 (1.0); control group: 6.6 (1.0)) days (*p* < 0.001).	10/11	Grade C—low
26	Iyengar et al. [43] United Kingdom	Controlled trial TKR = 174 or THR = 220	Early discharge with home based multidisciplinary team rehabilitation	Reduced hospital stay from 8.21 days (5–13) to 3.49 (0–11) for TKR and from 8.17 days (5‐15) to 2.84 days (0‐7) for THR	9/11	Grade C–low
27	Jones et al. (2011)[44] UK	Controlled trial TKR (Intervention = 322, control = 150)	Intervention: preoperative patient education program; control: conventional preoperative treatment	The mean length of stay significantly reduced from 7.0 ± 5.7 days (conventional group) to 5.0 ± 3.2 days (education group) (*p* < 0.01) and 20% more patients discharged early (within 1–4 days) in the education group.	8/11	Grade C—low
28	Khan et al. (2008)[45]	Systematic review TKR, THR	Multidisciplinary rehabilitation	Multidisciplinary rehabilitation programs can have a positive impact on service user‐related outcomes, such as function, quality or life, length of stay and cost	10/10	Grade A—high
29	Kim et al. (2003) [15]	Systematic review TKR, THR	Use versus the nonuse of clinical pathways following TKR or THR	Clinical pathways reduced length of stay and hospital costs and reduced or unchanged rates of complications.	10/10	Grade B—moderate
30	Larsen et al. (2008)[46] Denmark	Clinical trial (before–after) *N* = 247 (TKR or THR) (Intervention = 142, control = 105)	New accelerated perioperative care and rehabilitation procedures as provided by a new multidisciplinary organization	Intervention group significantly reduced hospital stay from 8.8 (3.0) days to 4.3 (1.8) days (*p* < 0.001). No significant differences in adverse effects	9/11	Grade C—low
31	Lin and Kaplan [47] United States	Cohort study (retrospective) TKR, THR *N* = 808 (TKR or THR)	Rehabilitation in acute rehabilitation facility postoperatively	Correlation between age and length of stay (LOS)—particularly for oldest group (*p* = 0.004) and gender (male *p* = 0.055), comorbid medical disease (*p* < 0.001) found to be predictive of increased LOS and marital status (single *p* = 0.001) and race (black *p* = 0.029) had influence in longer LOS	11/12	Grade C—low
32	Mak et al. [48]	Systematic review	Preoperative, perioperative and postoperative care	Multidisciplinary teams are necessary to optimize preparation for surgery.	9/10	Grade B—moderate
33	Malviya et al. [49] United Kingdom	Case‐control study TKR or THR ((Intervention=1,500(TKR=870), control=3,000 (TKR=1,632)))	Enhanced recovery program	The overall length of stay decreased from a mean of 8.5 to 4.8 days and from a median of 6 to 3 days (*p* < 0.001), resulting in a saving of 5418 bed days	10/11	Grade C—low
34	McDonald et al. [50]	Systematic review TKR or THR	Preoperative education	No significant difference in length of hospital stay between preoperative education and usual care (WMD −0.97; 95% CI −2.67 to 0.73). However, participants with more complex needs indicated that individually tailored programs of education and support were beneficial in reducing length of stay. There is evidence that preoperative education has a modest beneficial effect on preoperative anxiety	12/12	Grade A—high
35	McDonald et al. [51]	Systematic review TKR or THR	Preoperative education (the format of preoperative education ranged from one‐tone verbal communication, patient group sessions, or video or booklet with no verbal communication)	Length of hospital stay was statistically significantly reduced by almost 2 days in participants receiving preoperative education for knee replacement and education group showed lower scores in pain and not differ in function score at 12 months.	12/12	Grade A—high
36	Moyer et al. (2017)[52]	Systematic review and meta‐analysis TKR or THR	Preoperative exercise and education	Length of stay was significantly shorter in TKA group (SMD 5 0.54, 95% CI 5 0.24–0.84, *p*, 0.001) after preoperative program.	8/10	Grade A—high
37	Naylor et al. (2008)[53] Australia	Cohort study (prospective) *N* = 122 (TKR = 55, THR = 44)	Severe other joint disease or obesity	Participants with severe other joint disease recovered more slowly in terms of mobility than the nonsevere group (*p* = 0.005), also walked more slowly on the walk test and also had a greater chance of using a walking aid at 52 weeks (95% confidence interval)	7/11	Grade C—low
38	NICE [54] United Kingdom	NICE guideline TKR	Preoperative rehabilitation before and after surgery and postoperative rehabilitation	Preoperative rehabilitation for TKR or THR should include advice on exercises before and after surgery	N/A	Grade A—high
39	Nickinson et al. [55] United Kingdom	Cohort study (prospective) *N* = 56 (*T* *K* *R* = 29, *T* *H* *R* = 27)	Anxiety and depression levels	Females were more likely to become depressed than males odds ratio (OR) = 3.48 [95% CI: 1.01–11.88], mean time point for development of anxiety 1.94 days (most anxious at 2.47 days) and mean length of stay of 5 days for depressed or anxious service users (4 days for nonanxious/depressed patients)	9/12	Grade C—low
40	Pape et al. (2013)[56] Denmark	Case‐control study	A daily interprofessional meeting: surgeons, nurses, occupational therapists and physiotherapists used a checklist	A significant reduction in the length of stay in hospital in TKR (from a mean of 4.1 days (SD 2.1) to 2.7 days (SD 1.4), *p* < 0.05) but not in knee replacement participants.	11/11	Grade C—low
41	Pearson et al. [57] Australia	Case‐control study (retrospective) TKR *N* = 177 intervention = 119 control = 58	Clinical pathway (CP) management (OT participated)	A significant reduction in LOS in CP group (9 vs. 7 days; *p* < 0.0001) and a 66% increase in CP group who were admitted on the day of surgery (*p* < 0.0001) and a 19.6% increase in the number of patients discharged within 8 postoperative days (*p* < 0.01).	9/11	Grade C—low
42	Pennington et al. [58] New Zealand	Clinical trial TKR = 442 (intervention = 181, control = 261)	Clinical pathway (OT participated)	Length of stay reduced in intervention to 10.3 (3.4) days compared with 12.9 (4.9) in control group (*p* < 0.0001) and the percentage of patients discharged within 8 postoperative days rose from 23.8% in Group 1 to 62.8% in Group 2 (*p* < 0.0001).	9/11	Grade C—low
43	Sisak et al. (2019)[59] United Kingdom	Cohort study (retrospective, a single‐site) (TKR = 643, THR = 590)	Preoperative education	Patients with TKR attended the preoperative education class stayed a mean of 2.59 days less in hospital than those who did not attend (mean length of stay 4.52 (SD 1.26) vs. 7.11 days (SD 4.18) (95% CI −4.62, −0.54, p <0.02).	11/12	Grade C—low
44	Smith et al. (2015)[60]	Systematic review THR or TKR	Physical activity post‐THR and TKR	Following joint replacement service users have low expectations to participate in more activity than prior to surgery and limited evidence to suggest service users increase their activity levels postsurgery, but desired to regain the same level of physical activity levels prepathology.	9/10	Grade B—moderate
45	Soever et al. [61] Canada	Qualitative study *N* = 15 TKR or THR	Educational needs	Educational needs: knowing the team, condition information and management, access, preoperative activities, preparing for admission, rehabilitation process, functional recovery and follow‐up	8/10	Grade C—low
46	Specht et al. [62] Denmark	Qualitative study *N* = 8 (TKR or THR)	Patients′ experiences during the first 12 weeks after discharge in fast‐track	Patients appreciated only 1 or 2 days in hospital and they were not sufficiently involved in the discharge planning. There was a feeling of uncertainty and being left on their own after discharge, which could affect their pain management and recovery at home.	8/10	Grade C—low
47	Starks et al. [63] United Kingdom	Cohort study (prospective) *N* = 4193 (TKR = 2,344, THR = 1849)	Orthopedic enhanced recovery programs (ERP)	Length of stay was reduced 6 to 4 days after the introduction of ERP	10/12	Grade C—low
48	Stowers et al. [64] New Zealand	Mixed method: quasi‐experimental design study compared a prospective cohort (TKR or THR, ERAS = 100, control = 100)	Enhanced recovery after surgery (ERAS) program	Length of stay for patients (TKR and THR) was reduced by 1 day [4 (2) vs. 5 (2); *p* < 0.001]. (Control: June to August 2012; intervention: August to December 2013)	10/12	Grade C—low
49	Vissers et al. [65] Holland	Cohort study *N* = 65 (THR = 23, TKR = 42)	Physical function and actual daily activity	Actual daily activity had not increased between 6 months and 4 years postoperatively. Functional capacity and perceived physical function had increased in same time frame. Actual activities of daily living performance had not changed.	8/12	Grade C—low
50	Walter et al. (2007)[66] United States	Case‐control study TKR = 1680	Clinical pathways	Clinical pathway decreased the average length of stay for from 3.92 (average) to 2.98. ‐ Control 1 (2000): 3.94 (0.96) days, Control 2 (2001): 3.39 (1.13) days, Control 3 (2002): 3.33 (1.12) days ‐Intervention (2003): 2.98 (1.08) days	8/11	Grade C—low
51	Yoon et al. (2010)[67] United States	Cohort study (prospective) TKR or THR *N* = 261 (intervention = 163, control = 98)	Preoperative educational program	Education participants had a significantly shorter length of stay in THR (3.1 ± 0.8 days vs. 3.9 ± 1.4 days; *p* = 0.0001) and TKR (3.1 ± 0.9 days vs. 4.1 ± 1.9 days; *p* = 0.001)	10/12	Grade C—low

### 3.1. Improved ADL Functions

Improvements in ADL performance were primarily reported in cohort and case‐control studies, corresponding to low‐to‐moderate quality evidence (Grade C). Common effective strategies were comprehensive functional evaluations, use of standardized assessment tools, assistive device provision, and home environment modifications.

OT evaluations were described as comprehensive, addressing physical factors such as comorbidities, obesity, and presurgery functional status, as well as personal needs and goal setting [22, 47, 54]. The use of standardized assessment tools enabled accurate monitoring of functional performance throughout rehabilitation [21, 35]. Particularly, Alviar et al. [21] reported that patient‐reported outcome measures for arthroplasty rehabilitation should cover domains of activity, participation, and environment, as defined in the International Classification of Functioning, Disability and Health (ICF).

Assistive device provision and home environment advice were commonly described in observational studies, representing low‐quality evidence (Grade C) for associations with ADL outcomes. Multiple studies highlighted functional benefits from equipment training and home modification counseling [10, 22, 30, 40]. Typically, equipment provision training was delivered during preoperative rehabilitation [22, 30], whereas home modification consultations were incorporated into predischarge education or discharge planning [10].

### 3.2. Mental Health, Anxiety, Depressive Symptoms, and Cognition

Reductions in anxiety, depressive symptoms, and cognitive dysfunction were reported across a limited number of randomized trials and systematic reviews, reflecting low‐to‐moderate quality evidence (Grades B–C). Preoperative education and structured rehabilitation programs were most frequently reported in studies of moderate methodological quality (Grade B) and lower‐quality observational designs (Grade C).

A randomized controlled trial by Crowe and Henderson [30] reported lower presurgery anxiety in the pre‐rehabilitation group compared with usual care, as measured by the State Anxiety Inventory (40.0 vs. 45.7). Studies of fast‐track care models reported reductions in postoperative cognitive dysfunction of up to 8% [20]. Systematic reviews also reported that preoperative education programs were associated with reduced anxiety in patients undergoing hip or knee replacement [25, 50]. Reported OT educational interventions included provision of information and advice, home environment recommendations, and demonstrations of postsurgery exercises or assistive device use [9, 10, 25, 29, 34, 48, 51, 61, 62, 68].

### 3.3. Community Reintegration

Evidence addressing community reintegration following TKR was derived mainly from case studies and cohort designs, corresponding to low‐quality evidence (Grades C–D). Functional training, goal setting, and discharge planning were described primarily in lower‐grade evidence studies (Grades C–D), without consistent comparative designs.

Amini et al. [22] described a case in which planning for volunteering activities began during admission. Gillen et al. [35] reported that activities such as driving, appointment scheduling, and shopping were commonly targeted as part of community skills training. Physical activity was also examined as an outcome related to community reintegration. Vissers et al. [65] reported no significant increase in daily activity levels between 6 months and 4 years post‐TKR. In their systematic review, Smith et al. (2015) reported that physical activity levels following joint replacement remained low and that targeted physical activity interventions were discussed in relation to postsurgical activity outcomes.

Physical activity was also examined as an outcome related to community reintegration. Vissers et al. [65] reported no significant increase in daily activity levels between 6 months and 4 years post‐TKR.

Several studies described discharge planning and early referral to home‐based or community services as part of multidisciplinary care pathways. Iyengar et al. [43] reported that early discharge planning combined with home‐based multidisciplinary rehabilitation reduced hospital length of stay from 6.6 to 3.6 days.

### 3.4. Patient Satisfaction

Improvements in patient satisfaction were reported mainly in observational and descriptive studies, reflecting low‐quality evidence (Grade C). Reported OT‐related components included early goal setting, ADL training, and participation in multidisciplinary or fast‐track rehabilitation programs.

Amini et al. [22] and Gillen et al. [35] described improvements in patient‐reported satisfaction following individualized goal setting and tailored ADL interventions. Studies examining team‐based or fast‐track care pathways also reported higher patient satisfaction when OT was included as part of multidisciplinary rehabilitation [34, 37, 40].

### 3.5. Length of Hospital Stay

Reductions in hospital length of stay were consistently reported across randomized trials and systematic reviews of moderate to high quality (Grades A–B), as well as supported by cohort and case‐control studies (Grade C). Reported reductions in length of stay ranged from approximately 1 to 6 days across different healthcare systems and study designs.

A clinical trial conducted in New Zealand between 1995 and 1999 reported a decrease in hospital stay from 12.9 (4.9) days to 10.3 (3.4) days following the implementation of a clinical pathway [58]. In the United Kingdom, reductions in length of stay were reported from 6.6 to 3.6 days [42], from 8.21 to 3.49 days [43], from 8.5 to 4.8 days [49], from 7.8 to 6 days [32], and from 6 to 4 days [63]. In Canada, an enhanced recovery after surgery clinical trial conducted between 2017 and 2019 reported a reduction from 2.82 to 2.13 days [36]. Across these studies, occupational therapists were involved in preoperative education, functional training, and discharge planning as part of multidisciplinary teams.

Overall, evidence supporting reductions in hospital length of stay ranged from high‐quality systematic reviews and randomized trials (Grades A–B) to consistent findings from lower‐quality observational studies (Grade C).

## 4. Discussion

This systematic review, conducted as part of the “Developing Occupational Therapy Practice Guidelines for Patients with Total Knee Replacement” project, was designed to inform guideline development within the Korean healthcare system. It examined the effectiveness of OT interventions in enhancing perioperative care pathways in response to challenges such as population aging, rising hospital costs, and increasing demands for postoperative recovery. Unlike previous international reviews, this study adopted a context‐driven approach guided by stakeholder‐prioritized outcomes and feasibility within Korea′s clinical, cultural, and policy environment. This approach provides a framework for adapting international evidence to healthcare systems where OT services are underdeveloped or lack formal recognition.

Most included studies were rated as Grade C, reflecting the predominance of observational designs such as cohort and case‐control studies in OT research for TKR rehabilitation. Although fewer studies met Grade A or B criteria, findings across Grades A to C indicated beneficial roles of OT interventions across several outcome domains, although the strength of evidence varied by outcome. Importantly, lower‐grade evidence also offered contextually relevant insights. In healthcare systems such as Korea, where OT services for TKR rehabilitation remain underdeveloped, well‐documented case studies and practice‐based reports may provide practical guidance for service expansion and clinical decision‐making. For example, a Grade D case study described improvements in mobility, transfers, and self‐care following individualized OT intervention [22]. Although such findings have limited generalizability, they remain valuable for informing guideline development in settings with constrained high‐level evidence.

Having reviewed the overall quality and grading of the evidence, the following discussion synthesizes the core patterns of OT contributions identified across the included studies, focusing on their roles across the TKR care continuum. OT interventions were applied at multiple stages, including presurgical education and preparation, postsurgical ADL training, assistive device education, home environment modifications, and discharge planning. Among the OT interventions reviewed, several techniques consistently emerged as effective across multiple studies:

• ADL retraining and assistive device education functioned as core mechanisms for supporting functional independence and reducing caregiver burden, commonly implemented both before and after surgery [10, 22].
• Home environment modifications supported continuity of functional performance beyond hospital discharge by facilitating safer and more effective transitions to home settings [30, 40].
• Preoperative education programs addressed psychological readiness and expectation management, contributing to reduced perioperative distress and improved patient‐reported satisfaction [30, 51].
• Discharge planning by OT‐led teams supported system‐level efficiency by enhancing discharge readiness and coordination with community services within multidisciplinary care pathways [36, 43].

Although many interventions were delivered within multidisciplinary care pathways, several components were consistently led by occupational therapists. These OT‐led components included assessment of ADL, individualized ADL retraining, assistive device prescription and training, home environment modification, and assessment of functional readiness for discharge. Other elements, such as perioperative education, pain management, and early mobilization, were typically delivered collaboratively within multidisciplinary teams.

In addition to these functional and system‐level contributions, OT also addressed mental health and cognitive well‐being among patients undergoing TKR. Evidence suggests that OT interventions incorporating mental health assessment and personalized patient engagement may support psychological outcomes and contribute to overall recovery processes [28, 36, 55].

Building on the discussion of OT within integrated care, the literature also highlights the role of multidisciplinary clinical pathways in shaping postoperative outcomes following TKR. Since the early 2000s, coordinated care models have been introduced across various healthcare systems, with consistent evidence indicating more efficient inpatient care and earlier discharge [27, 42, 57, 58]. Across these pathway‐based models, the inclusion of OT has emerged as a key component of effective team functioning. Gleicher et al. [36] illustrated the system‐level impact of OT within an enhanced recovery program through a pre–post comparison. In this program, occupational therapists were initially absent from discharge planning but were later incorporated to address patients′ functional readiness for daily life. Following the inclusion of OT, the average hospital length of stay decreased from 2.82 to 2.13 days, accompanied by fewer emergency room visits. These findings suggest that OT involvement contributed to safer and more timely discharge processes by systematically assessing and addressing functional and participation‐related needs.

The magnitude of OT‐related benefits varies across healthcare systems. In several developed countries, coordinated care models have reduced average inpatient stays following TKR to fewer than 5 days [24, 33, 36, 64]. In contrast, the Korean healthcare system reports substantially longer hospitalizations, with an average length of stay of approximately 3 weeks [7]. This pronounced disparity emerged as a critical issue during the guideline development process, as length of stay is closely linked to healthcare costs and resource utilization [8]. In Korea, however, the integration of OT into orthopedic care pathways remains limited due to structural barriers, including restricted referral pathways, low reimbursement, and limited awareness of OT roles among healthcare professionals. Despite a growing OT workforce, these constraints have resulted in minimal OT involvement in orthopedic settings and prolonged hospital stays following joint replacement surgery [15]. In this context, multimodal enhanced recovery programs that integrate OT across the entire care pathway have been shown to be more effective in reducing hospital stay than surgical advances alone [4].

Although this review primarily focused on short‐term outcomes, the included literature also points to the potential longer‐term relevance of OT following TKR. Evidence suggests that limitations in physical activity and participation may persist years after surgery, highlighting the importance of continued support for community reintegration and self‐management [65]. OT‐supported activities related to daily participation and role resumption may therefore contribute to sustained functional independence and quality of life beyond hospital discharge [28, 35]. However, the durability of these effects remains insufficiently explored, underscoring the need for high‐quality longitudinal studies to better understand the long‐term impact of OT interventions after TKR.

Many countries have integrated OT into multidisciplinary clinical pathways, particularly within enhanced recovery programs, resulting in improved functional recovery and care transitions. The findings of this review are therefore relevant for healthcare systems at different stages of OT integration, including those where OT is not yet fully embedded in orthopedic rehabilitation. Although this review does not constitute a clinical guideline, it represents a foundational step toward future guideline development and provides an outcome‐driven framework that may inform more systematic integration of OT within the Korean healthcare context, where such models remain limited.

The included evidence supports key‐OT interventions that address gaps in continuity of care, including early ADL retraining, patient and caregiver education, home environment modification, and structured discharge planning. When incorporated into coordinated care pathways, these interventions may strengthen functional outcomes, enhance patient satisfaction, and support sustainable community reintegration following TKR. To translate these findings into practice, pilot programs integrating OT into TKR rehabilitation within the Korean healthcare system should be implemented and evaluated in terms of patient outcomes and cost‐effectiveness. In parallel, greater efforts are needed to support more rational and consistent referral to OT within orthopedic care, informed by evidence‐based practice guidelines. Systematic, guideline‐informed education and training for orthopedic teams and occupational therapists may further facilitate appropriate prescription, role clarity, and effective interprofessional collaboration in TKR rehabilitation.

### 4.1. Limitation

This systematic review has several limitations. First, the review was conducted with the specific objective of informing OT practice guidelines for TKR within the Korean healthcare system. Although this targeted focus enhances contextual relevance, it may limit the generalizability of findings to other healthcare systems or populations.

Second, although we utilized the PICO framework to guide study selection, this review was not designed to compare intervention effects between groups, but rather to identify and describe OT interventions applicable to TKR rehabilitation. Therefore, direct comparisons were not performed, as the purpose of this review was to map suitable interventions rather than evaluate their relative effectiveness.

Third, the literature search included studies published between 2000 and 2021, which may have excluded earlier relevant research as well as studies from the most recent 4 years. In addition, only studies published in English were included, which may have introduced language and publication bias and resulted in the omission of relevant evidence published in other languages or grey literature. Practice‐based reports or nonpeer‐reviewed sources that may be informative in certain healthcare contexts were therefore not captured. Nevertheless, the core OT intervention domains identified in this review remain relevant to the current Korean context.

Additionally, several included studies were of lower methodological quality and small sample sizes. These were retained in order to reflect the full scope of available OT evidence for TKR, particularly given the scarcity of high‐level research in this area. Nonetheless, the inclusion of lower‐grade evidence may weaken the overall strength of the conclusions.

Furthermore, the evolving nature of OT practices and the dynamic healthcare landscape may also introduce variables that were not fully captured in this review. Thus, while providing valuable insights for the Korean healthcare context, our findings should be interpreted with caution and an understanding of these limitations when considering their application to more diverse healthcare settings.

## 5. Conclusion

This systematic review demonstrates that OT plays a meaningful role in TKR rehabilitation by supporting functional recovery, improving ADL, and addressing mental and cognitive well‐being. Evidence from international care models indicates that when OT is integrated into multidisciplinary, coordinated pathways, recovery efficiency is improved and transitions from hospital to home are better supported.

In the Korean healthcare context, where post‐TKR hospitalization remains relatively prolonged, these findings highlight an opportunity to enhance rehabilitation pathways through more systematic integration of OT. Future research should focus on evaluating OT‐inclusive rehabilitation models, including pilot initiatives and longitudinal studies, to better understand their clinical and economic impact.

Overall, this review suggests that OT contributes to TKR rehabilitation, with relatively stronger evidence supporting reductions in hospital length of stay, whereas evidence for improvements in ADL performance, mental health, and community participation remains less robust.

## Funding

No funding was received for this manuscript.

## Conflicts of Interest

The authors declare no conflicts of interest.

## Data Availability

The data that support the findings of this study are available from the corresponding author upon reasonable request.
